# Bevacizumab as maintenance therapy in mCRC: Interpreting results of the MOMA trial

**DOI:** 10.18632/oncotarget.26870

**Published:** 2019-04-19

**Authors:** Federica Marmorino, Alfredo Falcone, Chiara Cremolini

**Affiliations:** Chiara Cremolini: Unit of Medical Oncology, Azienda Ospedaliero-Universitaria Pisana, Department of Translational Research and New Technologies in Medicine and Surgery, University of Pisa, Pisa, Italy

**Keywords:** maintenance, metastatic colorectal cancer, bevacizumab

The concept of maintenance rises from the objective to limit the duration of the upfront combination therapy to a short induction phase able to achieve disease control, followed by a less toxic treatment able to delay disease progression, while improving patients’ quality of life. These objectives are especially relevant with upfront intensive regimens, such as FOLFOXIRI/bevacizumab that is recognized by major international guidelines as a valuable first-line option for selected mCRC patients.

Drawing from this rationale, the phase II randomized MOMA study was designed to assess the efficacy of a shortened induction phase of FOLFOXIRI/bevacizumab, limited to 8 instead of 12 cycles as in the previous TRIBE trial [1], followed by two different maintenance strategies: bevacizumab alone or bevacizumab plus metronomic capecitabine and cyclophosphamide [2].

The rationale for using metronomic chemotherapy (metroCT) in the maintenance phase was based on the synergistic anti-angiogenic effect of the frequent administration of low doses of cytotoxic agents with the anti-VEGF-A bevacizumab, demonstrated in preclinical models [3]. Moreover, while during treatment breaks between cycles of conventional antineoplastic agents, endothelial cells get a chance to recover, this effect may be overcome by the metronomic approach. Finally, a previous experience of our group showed signals of activity of low doses of uracil-tegafur (UFT) and cyclophosphamide plus celecoxib, an agent involved in angiogenesis inhibition, in gastrointestinal malignancies, at the price of a very favourable toxicity profile [4].

MOMA trial did not meet its primary endpoint of demonstrating prolonged progression-free survival (PFS) with the addition of metroCT to bevacizumab during maintenance; median PFS was not different between the two arms (10.3 months in metroCT arm plus bevacizumab and 9.4 months in bevacizumab arm, HR: 0.94, *p* = 0.680). No difference in terms of overall survival (OS) was reported: median OS durations were 22.5 and 28 months in metroCT plus bevacizumab and bevacizumab arm, respectively (HR: 1.16, *p* = 0.336). In the overall population, the RECIST response rate with FOLFOXIRI/bevacizumab was 63%.

When looking at trial’s design, the choice of a suboptimal control arm immediately leaps out: today bevacizumab alone cannot be regarded as a “standard” maintenance strategy [5]. However, when the MOMA study was conceived, this choice was driven by the intent to offer a chemotherapy-free interval after the induction phase, in the absence of results from randomized trials that failed to demonstrate a significant impact of bevacizumab alone over treatment holiday [6, 7].

Differently from positive findings reported in other solid malignancies, these negative results achieved with metroCT confirm those previously reported in another phase II single-arm prospective trial where no response was observed with the combination of metronomic doses of capecitabine and cyclophosphamide in a population of chemorefractory mCRC patients [8, 9]. Therefore, based on the current amount of knowledge, mCRC seems a hostile field for the application of the metronomic approach, probably due to the intrinsic aggressiveness of this disease.

Putting results from the MOMA study in the frame of other available evidence with the upfront use of FOLFOXIRI/bevacizumab, consistent data were reported in terms of safety and activity, though in a population at high prevalence of poor prognostic features and with a shortened duration of the induction phase (four instead of six months). Conversely, shorter PFS and OS durations were reported than in the FOLFOXIRI/bevacizumab arm of the phase III TRIBE trial, where 12.1 and 31.0 months of median PFS and OS were achieved [1].

Though acknowledging the methodological inappropriateness of such a comparison, two considerations might explain this difference:

1) the shorter duration of the induction chemotherapy;

2) the suboptimal maintenance regimens (bevacizumab alone or plus metroCT *versus* bevacizumab plus standard dose 5FU/LV).

Another phase III randomized trial in the first- and second-line treatment of mCRC was recently conducted in Italy by the GONO (Gruppo Oncologico del Nord Ovest) Group, named TRIBE2 [10]. 679 patients were randomized to receive FOLFOX/bevacizumab followed by FOLFIRI/bevacizumab after disease progression (PD) or FOLFOXIRI/bevacizumab followed by the reintroduction of the same regimen after PD. Combination treatments were administered up to 8 cycles, followed by 5-FU/LV plus bevacizumab until PD. While OS results are not mature yet, median PFS with FOLFOXIRI/bevacizumab was 12.0 months, thus suggesting a relevant impact of 5FU/LV plus bevacizumab maintenance in extending the duration of PFS, as well as the oxaliplatin- and irinotecan-free intervals, thus making more feasible and efficacious treatments after progression.

Taken together, these findings support bevacizumab plus standard dose 5FU/LV as the preferable maintenance option following first-line FOLFOXIRI/bevacizumab and underline its role in the therapeutic strategy of mCRC patients to optimize the efficacy of first-line regimens.
